# Genomic characterization and biological activities of cell-free supernatant from *Lactiplantibacillus pentosus* CROT01 against clarithromycin-resistant *Helicobacter pylori*

**DOI:** 10.3389/fcimb.2026.1882551

**Published:** 2026-08-12

**Authors:** Chonticha Romyasamit, Morteza Saki, Phanvasri Saengsuwan, Phuvamin Suriyaamporn, Teeratas Kansom, Theerachai Thammathiwat, Wuttapon Sadaeng, Alisa Thiamsakul, Natthapong Sueviriyapan, Kitbordin Thongduang, Kannaphak Tantasith, Raksiri Kaewtawee, Phoomjai Sornsenee

**Affiliations:** 1 Department of Medical Technology, School of Allied Health Sciences, Walailak University, Nakhon Si Thammarat, Thailand; 2 Research Center for Microbiome, Systems Biology, and Medical Innovation, Walailak University, Nakhon Si Thammarat, Thailand; 3 Research Center in Tropical Pathobiology, Walailak University, Nakhon Si Thammarat, Thailand; 4 Department of Microbiology, Faculty of Medicine, Ahvaz Jundishapur University of Medical Sciences, Ahvaz, Iran; 5 Department of Biomedical Sciences and Biomedical Engineering, Faculty of Medicine, Prince of Songkla University, Songkhla, Thailand; 6 Research and Innovation Center for Advanced Therapy Medicinal Products, Division of Industrial Pharmacy, Faculty of Pharmacy, Silpakorn University, Nakhon Pathom, Thailand; 7 Division of Nephrology, Department of Medicine, Faculty of Medicine, Naresuan University, Phitsanulok, Thailand; 8 Department of Oral Diagnosis, Faculty of Dentistry, Naresuan University, Phitsanulok, Thailand; 9 Institute of Marine Science, Burapha University, Chonburi, Thailand; 10 The Petroleum and Petrochemical College, Chulalongkorn University, Bangkok, Thailand; 11 Faculty of Public Health, Naresuan University, Phitsanulok, Thailand; 12 Faculty of Law, Naresuan University, Phitsanulok, Thailand; 13 School of Architecture and Design, Walailak University, Nakhon Si Thammarat, Thailand; 14 Department of Family and Preventive Medicine, Faculty of Medicine, Prince of Songkla University, Songkhla, Thailand

**Keywords:** anti-bacterial activity, *Helicobacter pylori*, *Lactiplantibacillus pentosus*, postbiotic, whole genome sequencing

## Abstract

*Helicobacter pylori* is a major gastric pathogen associated with chronic gastritis, peptic ulcers, and gastric cancer, while increasing antibiotic resistance has reduced the effectiveness of standard eradication therapies. This study investigated the antibacterial, antibiofilm, anti-inflammatory, probiotic-related, and genomic properties of cell-free supernatant (LCFS) derived from *Lactiplantibacillus pentosus* CROT01 against *H. pylori*. LCFS exhibited inhibitory activity against all tested *H. pylori* strains, including the clarithromycin-resistant strain ATCC 700684, with inhibition zones ranging from 10.67 ± 0.58 to 17.33 ± 1.53 mm. The minimum inhibitory concentration (MIC) and minimum bactericidal concentration (MBC) ranged from 12.5–50 mg/mL and 25–50 mg/mL, respectively. LCFS significantly inhibited biofilm formation and eradicated established *H. pylori* biofilms. Scanning electron microscopy revealed marked morphological alterations in treated cells, including membrane disruption, deformation, and cellular shrinkage. Anti-inflammatory activity was demonstrated through suppression of nitric oxide production in lipopolysaccharide-stimulated RAW 264.7 cells. In addition, *L. pentosus* CROT01 exhibited tolerance to acidic pH and pancreatin, high cell surface hydrophobicity, and moderate adhesion ability toward Caco-2 cells. Whole-genome sequencing identified several bacteriocin-associated genes, including *plantaricin E*, *plantaricin F*, *pediocin, enterolysin A*, and *bovicin-related peptides*, whereas no virulence-associated or acquired antimicrobial resistance genes were detected. Thus, these findings suggest that LCFS derived from *L. pentosus* CROT01 possesses promising anti-*H. pylori* activity and may represent a potential postbiotic-based strategy against antibiotic-resistant *H. pylori* infection.

## 1 Introduction

*Helicobacter pylori* is a Gram-negative, helical, microaerophilic bacterium that colonizes the human gastric mucosa and represents one of the most prevalent chronic infections worldwide (Graham, 2024; Duan et al., 2025). It is estimated that more than 50% of the global population, corresponding to over 4.4 billion individuals, is infected with *H. pylori*, with prevalence rates exceeding 70% in developing regions characterized by inadequate sanitation and limited access to healthcare (Akinpelu et al., 2025). In Southeast Asia, particularly Thailand, the prevalence ranges from approximately 40% to 60%, with higher rates observed in rural and underserved populations (Uchida et al., 2015). Persistent colonization is closely associated with a spectrum of gastrointestinal diseases, including chronic gastritis, peptic ulcer disease, mucosa-associated lymphoid tissue lymphoma, and gastric cancer (Salvatori et al., 2023; Duan et al., 2025). *H. pylori* has been classified as a Group 1 carcinogen by the International Agency for Research on Cancer (Cancer IAfRo, 1994). Despite extensive research and the availability of established antibiotic eradication regimens, *H. pylori* infection remains a significant global health challenge.

*H. pylori* pathogenesis is multifactorial, involving complex interactions between bacterial virulence factors and host immune responses. Key mechanisms include urease-mediated neutralization of gastric acid, which enables bacterial survival in the highly acidic gastric environment, and flagella-driven motility, which facilitates penetration of the mucus layer and colonization of the gastric epithelium (Sharndama and Mba, 2022). Major virulence determinants, such as cytotoxin-associated gene A (CagA) and vacuolating cytotoxin A (VacA), disrupt epithelial integrity, induce cellular damage, and modulate host signaling pathways (Cheok et al., 2021). These processes trigger a persistent inflammatory response characterized by the release of pro-inflammatory mediators, including nitric oxide (NO), interleukin (IL), and tumor necrosis factor-alpha (TNF-α), which contribute to mucosal injury and disease progression. Sustained inflammation not only promotes bacterial persistence but also increases the risk of malignant transformation (Cadamuro et al., 2014).

The standard management of *H. pylori* infection typically involves triple therapy consisting of clarithromycin, a proton pump inhibitor (PPI), and either amoxicillin or metronidazole (Aumpan et al., 2023). However, treatment efficacy has declined substantially due to the increasing emergence of clarithromycin-resistant strains, primarily driven by point mutations in the 23S rRNA gene that reduce antibiotic binding (Hasanuzzaman et al., 2024). Owing to rising eradication failure rates, clarithromycin-resistant *H. pylori* has been classified by the World Health Organization as a high-priority pathogen (Kocsmár et al., 2021). Furthermore, conventional antibiotic therapies may disrupt the gut microbiota, promote antimicrobial resistance (AMR), and result in adverse effects, recurrence, and reduced patient compliance (Alaoui Mdarhri et al., 2022). These limitations highlight the urgent need for safe and effective alternative therapeutic strategies.

Probiotics are live microorganisms that when administered in adequate amounts, confer a health benefit on the host (Ma et al., 2023). They have been of much interest as alternative strategies for the management of gastrointestinal diseases including *H. pylori* infection. The most common probiotic groups are *Limosilactobacillus* (previous name: *Lactobacillus*) spp., *Bifidobacteria*, *Streptococcus* spp., *Pediococcus* spp., *Propionibacterium* spp., and yeasts, many of which possess antimicrobial and immunomodulatory properties (Wala et al., 2023). Various probiotic strains such as *L. acidophilus*, *L. rhamnosus* GG, *L. gasseri* OLL2716, *L. reuteri*, *L. plantarum* have demonstrated inhibitory effects against *H. pylori* (Ji and Yang, 2020). These effects are exerted through various mechanisms such as organic acids and bacteriocins production, urease activity inhibition, competitive exclusion, interference with bacterial adhesion and modulation of inflammatory responses (Ji and Yang, 2020). However, the use of live probiotics continues to be limited by issues of viability, stability during storage and gastrointestinal passage, and possible risk of infection in immune-compromised individuals (Sarita et al., 2024). The efficacy of probiotics is often strain specific and is influenced by environmental factors that make it difficult to reproduce and standardize (Sarita et al., 2024). This has led to an increased interest in postbiotics, defined as non-viable microbial products or metabolites from probiotic microorganisms (Kumar et al., 2024). Postbiotics have several beneficial properties of their parental strains such as antimicrobial, antibiofilm, antioxidant and anti-inflammatory activities (Kumar et al., 2024). They have better stability, safety and ease of standardization.

Among probiotic candidates, species within the genus *Lactiplantibacillus*, particularly *L. pentosus*, have attracted considerable attention due to their remarkable functional properties and adaptability to diverse environments (Thakur et al., 2024; Chen et al., 2025). These bacteria are known to produce a wide range of bioactive compounds, including organic acids, bacteriocins, and exopolysaccharides, which contribute to their antimicrobial and immunomodulatory activities (Chen et al., 2025). Environmental niches such as sediment ecosystems remain largely underexplored reservoirs of microbial diversity and may provide valuable sources for the isolation of novel strains with unique functional characteristics (Chen et al., 2006).

The present study aimed to isolate and characterize *L. pentosus* CROT01 from sediment-derived samples and to evaluate its potential as a postbiotic-based therapeutic candidate against clarithromycin-resistant *H. pylori*. In addition, genomic analysis was integrated with functional assays to investigate its antibacterial and anti-inflammatory activities, including the modulation of nitric oxide production in lipopolysaccharide-stimulated macrophages.

## 2 Materials and methods

### 2.1 Bacterial strains and growth conditions

Two reference strains *H. pylori* ATCC 700684 and *H. pylori* ATCC 43504 were purchased from American Type Culture Collection (ATCC, Manassas, VA, USA). Two clinical *H. pylori* isolates (004 and 117) were recovered from gastric biopsy specimens obtained from Songklanagarind Hospital. The isolates were previously identified by conventional microbiological methods, MALDI-TOF MS, and whole-genome sequencing, and their antimicrobial susceptibility profiles and virulence characteristics have been described previously (Romyasamit et al., 2025). All strains were inoculated on blood agar (BA) (HiMedia, Mumbai, India) with 7% horse blood at 37 °C for 48 h in microaerophilic conditions. After incubation, the bacterial colonies were inoculated into brain heart infusion (BHI) broth (HiMedia) and further cultured at 37 °C for 24 h. The bacterial strains were stored at −80 °C in BHI broth containing 30% glycerol for long-term storage until further use in experiments.

### 2.2 Collection of sediment samples and isolation

Sediment samples were collected from the Mekong River in Nong Khai Province, Thailand, and transported to the laboratory under sterile conditions for microbiological analysis. Lactic acid bacteria (LAB) were isolated by culturing the samples on de Man, Rogosa, and Sharpe (MRS) agar (HiMedia) followed by incubation at 37 °C for 48 h. Distinct colonies were subsequently streaked onto fresh MRS agar plates and incubated under identical conditions for an additional 24 h to obtain pure cultures. The selected isolate, designated *L. pentosus* CROT01, was maintained in MRS broth (HiMedia) supplemented with 30% (v/v) glycerol and preserved at −80 °C for subsequent analyses.

### 2.3 Inhibition of *H. pylori*

The antimicrobial activity of isolated bacteria against *H. pylori* was evaluated by agar well diffusion assay according to by Sornsenee, et al (Sornsenee et al., 2024). with slight modifications. *H. pylori* strains were standardized to 0.5 McFarland and spread on BA supplemented with 7% horse blood. Wells were prepared aseptically (diameter 6 mm) and 100 µL of isolated bacteria culture was added to each well, MRS broth was used as negative control. Plates were incubated at 37 °C for 48 h under microaerophilic conditions. Antimicrobial activity was determined by the presence of inhibition zones larger than the diameter of the well. All experiments were performed in triplicate, and the results were expressed as mean ± standard deviation (SD).

### 2.4 Phenotypic and MALDI-TOF identification

Gram staining, microscopic examination, and catalase activity were performed according to Bergey’s Manual of Systematic Bacteriology (Paul De Vos, 2009). Bacterial identification was further confirmed using matrix-assisted laser desorption/ionization time-of-flight mass spectrometry (MALDI-TOF MS) with the BioTyper system (Bruker Daltonics, Karlsruhe, Germany) following the manufacturer’s instructions. Briefly, fresh bacterial colonies were subjected to ethanol-formic acid extraction, mixed with α-cyano-4-hydroxycinnamic acid (HCCA) matrix solution, and analyzed using a Bruker Ultraflex III TOF/TOF mass spectrometer in positive linear mode over a mass range of 2–20 kDa. Spectral data were processed using BioTyper 2.0 software.

### 2.5 Characterization of probiotic properties

#### 2.5.1 Tolerance to pepsin, pancreatin, and bile salt

The tolerance of *L. pentosus* CROT01 to simulated gastrointestinal conditions was evaluated based on its tolerance to pepsin, pancreatin, and bile salts according to the method of Monteagudo-Mera et al. (2012) with slight modifications. Pepsin and pancreatin solutions were prepared in MRS broth at concentrations of 3 g/L and 1 g/L, with pH adjusted to 2.0 and 8.0, respectively. Overnight bacterial cultures were harvested by centrifugation at 5,000 × g for 5 min, washed with phosphate-buffered saline (PBS), and resuspended in the respective enzyme-containing media. The suspensions were incubated at 37 °C for 3 h in pepsin solution and 4 h in pancreatin solution. Viable bacterial counts before and after treatment were determined on MRS agar plates, and survival rates were subsequently calculated.

For bile salt tolerance, bacterial cells were collected by centrifugation and resuspended in MRS broth supplemented with 0.3% (w/v) bile salts (Sigma-Aldrich, St. Louis, MO, USA). Following incubation at 37 °C for 4 h, viable colonies were enumerated on MRS agar plates, and the percentage of surviving cells was calculated accordingly.


Survival rate (%) = [Final (Log CFU/mL)]/[Initial (Log CFU/mL)] × 100

#### 2.5.2 Auto-aggregation assay

The auto-aggregation ability of *L.pentosus* CROT01 was evaluated by the method of Sornsenee, et al (Sornsenee et al., 2026). with minor modifications. Briefly, overnight bacterial cultures were pelleted at 5,000 × g for 5 min and washed twice in phosphate-buffered saline (PBS, pH 7.2). The cell pellets were re-suspended in the same buffer and incubated at 37 °C under anaerobic conditions. The upper suspension optical density (OD) was measured at 600 nm at 0, 2, 4 and 24 h. The ratio of auto-aggregation was calculated using the following equation:


Auto-aggregation(%) = [1 − (Atime/A0)] × 100

where Atime is the absorbance at a given time point and A0 is the initial absorbance at 0 h.

#### 2.5.3 Cell surface hydrophobicity assay

Cell surface hydrophobicity of *L. pentosus* CROT01 was determined using the xylene partitioning method described by Krausova et al. (2019), with slight modifications. Briefly, bacterial cells were harvested, washed, and resuspended in phosphate-buffered saline (PBS, pH 7.2) to obtain an initial absorbance (A_0_) of approximately 0.5 at 600 nm. Three milliliters of the bacterial suspension was mixed with 1 mL of xylene, vortexed for 2 min, and allowed to stand at room temperature for 30 min to permit phase separation. The aqueous phase was carefully collected, and the absorbance (A) was measured at 600 nm. Cell surface hydrophobicity was calculated using the following equation:


$$\text{H\% = }\left[\left({\text{A}}_{0}{\text{ - A}}_{0}\right){\text{/A}}_{0}\right]\times 100$$

where A_0_ and A represent the absorbance values before and after xylene extraction, respectively”.

#### 2.5.4 Adhesion to the Caco-2 cell line

The adhesion abilities to *L. pentosus* CROT01 was evaluated with human colorectal adenocarcinoma (Caco-2) cells purchased from ATCC following the method of Zawistowska-Rojek et al. (2022) with minor modifications. Caco-2 cells were cultured in Dulbecco’s Modified Eagle Medium (DMEM; Thermo Fisher Scientific, Waltham, MA, USA) with 3 mM L-glutamine and antibiotics added and incubated at 37 °C in a humidified atmosphere with 5% CO_2_ until confluence of 85–90%. Each well was filled with bacterial suspensions (10^8^ CFU/mL), and incubated for 2 h. The monolayers were washed with sterile phosphate-buffered saline (PBS) to remove non-adherent bacteria and then detached with 2.5% (w/v) trypsin solution. The released bacteria were serially diluted and counted on MRS agar plates. The adhesion ability (%) was calculated with the equation:


$$\%\text{ adhesion ability }=\;\left({\text{V}}_{1}* 100\right){\text{/V}}_{0}$$

where V_0_ is initial viable bacteria and V_1_ is the viable count of bacteria attached to Caco-2 cells after incubation.

#### 2.5.5 Hemolytic activity

The hemolytic activity of *L. pentosus* CROT01 was evaluated on BA plates according to the method of Leite et al. (2015). After incubation at 37 °C for 24 h, hemolytic activity was determined based on the presence of α-, β-, or γ-hemolytic zones surrounding the bacterial colonies.

#### 2.5.6 Antibiotic susceptibility

Antibiotic susceptibility testing was performed following the guidelines of the Clinical and Laboratory Standards Institute (CLSI, 2025) (CLSI, 2019). Antibiotic susceptibility testing was performed by the disc diffusion method (Oxoid, Hampshire, UK) with ampicillin (10 µg), vancomycin (30 µg), gentamicin (10 µg), erythromycin (15 µg), clindamycin (2 µg), tetracycline (30 µg), kanamycin (30 µg), chloramphenicol (30 µg), and streptomycin (10 µg). The plates were incubated at 37 °C for 24 h and the diameters of the inhibition zones were measured in mm. *L. pentosus* CROT01 were categorized as susceptible, intermediate, or resistant based on CLSI interpretive criteria.

### 2.6 Determination of MIC and MBC

#### 2.6.1 Preparation of lyophilized cell-free supernatants

LCFS were prepared according to the method described by Sornsenee et al. (2021), with slight modifications. *L. pentosus* CROT01 was cultured in 1,000 mL of MRS broth and incubated at 37 °C for 24 h under anaerobic conditions. The bacterial cultures were centrifuged at 10,000 × g, and the supernatants were subsequently sterilized using a 0.2 µm membrane filter (Sigma, Steinheim, Germany). Sterile CFSs and MRS broth as a control were stored at −80 °C for 24 h prior to lyophilization. Freeze-drying was performed using a lyophilizer (Lyophilization Systems Inc., USA) at −40 °C to −30 °C under 0.2 mbar pressure for 24 h. The lyophilized powders were preserved at −20 °C and reconstituted in sterile deionized water before use.

#### 2.6.2 Minimum inhibitory concentration and minimum bactericidal concentration of *L. pentosus* CROT01

The antimicrobial activity of LCFS against *H. pylori* were determined using the broth microdilution method in 96-well plates according to the Clinical and Laboratory Standards Institute (CLSI, 2022) guidelines (CLSI, 2022). Serial dilutions of LCFS (200–1.56 mg/mL) were prepared in Mueller–Hinton broth (MHB) (HiMedia, Mumbai, India). The reported concentrations represent the dry weight of the LCFS. Bacterial suspensions adjusted to McFarland standard 2 were inoculated into each well and incubated at 37 °C for 72 h under microaerophilic conditions. Minimum inhibitory concentration (MIC) was determined using resazurin as a viability indicator. Following incubation, 20 µL of 0.01% (w/v) resazurin solution prepared in sterile PBS was added to each well and further incubated for 4 h. The MIC was defined as the lowest concentration of LCFS that prevented the color change from blue to pink, indicating inhibition of bacterial growth.

MBC was determined by subculturing samples from wells showing no visible growth onto Brucella agar supplemented with 7% horse blood. After incubation at 37 °C for 72 h under microaerophilic conditions, bacterial colonies were enumerated. The MBC was defined as the lowest LCFS concentration resulting in complete inhibition of colony formation.

#### Scanning electron microscopy

The effects of LCFS from *L. pentosus* CROT01 on the morphology of *H. pylori* cells were evaluated by SEM according to Kim et al. (2003) Briefly, *H. pylori* ATCC 700684 was cultivated MHB at 37 °C for 48 h. Bacterial suspensions were adjusted to 2 McFarland and placed in a centrifuge tube with 4X MIC of LCFS and incubated at 37 °C for 24 h under microaerophilic conditions. MRS broth served as a negative control. Then the samples were centrifuged at 10,000 rpm for 5 min and washed with PBS and centrifuged. Control and LCFS-treated cells were dropped onto a sterile glass coverslip (0.5 cm × 0.5 cm), air dried. All samples were fixed with 2.5% (v/v) glutaraldehyde (Sigma Aldrich, St Louis, MO, USA) in 0.1 M phosphate buffer for 24 h at 4 °C. Cells were then dehydrated in a graded ethanol series (40%, 60%, 80%, and 95% v/v) for 15 min each session followed by two dehydration steps in 100% ethanol (Thermo Fisher Scientific, NY, USA) for 15 min followed by gold coating. The morphology of bacteria after treatment with LCFS was observed using a field emission scanning electron microscope (Oxford Instruments, Quanta, Japan).

### 2.7 Biofilm inhibition assay

The antibiofilm activity of LCFS from *L. pentosus* CROT01 against *H. pylori* was evaluated using a crystal violet microtiter plate assay adapted from the method described by Sornsenee et al. (2021), with slight modifications. Briefly, overnight cultures of *H. pylori* were adjusted to 2 McFarland in MHB and inoculated into sterile 96-well microplates containing LCFS at concentrations corresponding to 0.5× MIC and 1× MIC. The plates were incubated at 37 °C for 72 h under microaerophilic conditions to allow biofilm formation. Following incubation, the culture medium was carefully removed, and the wells were gently washed three times with PBS (pH 7.4) to eliminate non-adherent cells. The attached biofilms were fixed with 200 µL of 99% methanol for 15 min and stained with 200 µL of 0.1% (w/v) crystal violet solution for 10 min at room temperature. Excess stain was removed by rinsing the wells four times with distilled water. The stained biofilms were subsequently solubilized using 95% ethanol, and the absorbance was measured at 570 nm using a microplate reader. All experiments were performed in triplicate. The percentage of biofilm inhibition was calculated using the following equation:

Biofilm inhibition (%) = [(OD 570 of control well − OD 570 of treated well)/OD 570 of control well] × 100.

### 2.8 Biofilm eradication assays

The biofilm eradication activity of LCFS from *L. pentosus* CROT01 against preformed *H. pylori* biofilms was evaluated according to the method described Sornsenee et al. (2021), with slight modifications. Briefly, overnight cultures of *H. pylori* adjusted to 2 McFarland standard were inoculated into sterile 96-well microplates and incubated at 37 °C for 72 h under microaerophilic conditions to allow mature biofilm formation. Following incubation, the wells were gently washed with PBS (pH 7.4) to remove non-adherent cells. Preformed biofilms were then treated with LCFS at concentrations corresponding to 0.5× MIC and 1× MIC and further incubated at 37 °C for 24 h under microaerophilic conditions. After treatment, the wells were washed three times with PBS and stained with 0.1% (w/v) crystal violet solution as previously described. The stained biofilms were solubilized in 95% ethanol, and absorbance was measured at 570 nm using a microplate reader. All experiments were performed in triplicate. The percentage of biofilm eradication was calculated using the following equation:

Biofilm eradication (%) = [(OD 570 of control well − OD 570 of treated well)/OD 570 of control well] ×100.

### 2.9 Cell lines and culture conditions

RAW 264.7 macrophage cells (TIB-71) and AGS human gastric adenocarcinoma epithelial cells (ATCC CRL-1739) were obtained from the American Type Culture Collection (ATCC, Manassas, VA, USA). AGS cells were cultured in Ham’s F-12 medium (Gibco, Thermo Fisher Scientific, Waltham, MA, USA) supplemented with 10% fetal bovine serum (FBS; Gibco) and 1% penicillin–streptomycin solution at 37 °C in a humidified atmosphere containing 5% CO_2_. Cells were subcultured and maintained at approximately 90% confluency.

RAW 264.7 cells were cultured in Dulbecco’s Modified Eagle’s Medium (DMEM; Gibco, Thermo Fisher Scientific) supplemented with 10% FBS and 1% penicillin–streptomycin solution at 37 °C under 5% CO_2_. The cells were routinely subcultured and maintained at 80–90% confluency prior to experiments.”.

### 2.10 Cytotoxicity assay

The cytotoxic effects of LCFS from *L. pentosus* CROT01 on AGS and RAW 264.7 cells were evaluated using the MTT assay as previously described (Sornsenee et al., 2021), with slight modifications. Briefly, AGS and RAW 264.7 cells were seeded into 96-well plates at a density of 5 × 10^4^ cells/mL and incubated at 37 °C in a humidified atmosphere containing 5% CO_2_. The cells were subsequently treated with LCFS at concentrations ranging from 0.3125 to 20 mg/mL for 24 h. Following treatment, the supernatants were discarded, and the cells were washed with PBS. Subsequently, 100 µL of MTT solution (0.5 mg/mL in PBS; Sigma-Aldrich, St. Louis, MO, USA) was added to each well and incubated in the dark for 3 h. The resulting formazan crystals were dissolved in 200 µL of dimethyl sulfoxide (DMSO; Sigma-Aldrich), and absorbance was measured at 570 nm using a microplate reader. All experiments were performed in triplicate.


Cell viability (%) = (Atest/Acontrol) × 100

where Atest represents the absorbance of LCFS-treated cells and Acontrol represents the absorbance of untreated control cells.

### 2.11 Nitric oxide production inhibition assay

This assay was performed according to the method of Khanna et al. (2020) with slight modifications. Briefly, RAW 264.7 cells were seeded into 96-well plates and treated with LCFS from *L. pentosus* CROT01 at LC_5_ and LC_10_ concentrations in the presence or absence of 20 ng/mL lipopolysaccharide (LPS) (Sigma-Aldrich, St. Louis, MO, USA). Cells treated with LPS alone served as the positive control. After incubation at 37 °C in a humidified atmosphere containing 5% CO_2_ for 24 h, nitric oxide (NO) production was determined by mixing equal volumes of culture supernatant and Griess reagent (Sigma-Aldrich) followed by incubation at 37 °C for 30 min. Absorbance was measured at 570 nm using a microplate reader. All experiments were performed in triplicate.

Nitric oxide production = (ODtest/ODstandard) × concentration of standard.

### 2.12 DNA extraction and genomic analysis

Genomic DNA of *L. pentosus* CROT01 was extracted using the DNeasy Blood & Tissue Kit (QIAGEN, Hilden, Germany) according to the manufacturer’s instructions. DNA quality and concentration were evaluated spectrophotometrically based on the A260/A280 ratio and further confirmed by agarose gel electrophoresis. Whole-genome sequencing (WGS) was performed at the Beijing Genomics Institute (BGI, China) using the MGISEQ-2000 platform with 150 bp paired-end sequencing reads.

Raw sequencing reads were analyzed using the BacSeq v1.0 pipeline (Chukamnerd et al., 2023), which integrates multiple bioinformatics tools for genome assembly, annotation, and quality assessment. Genome assembly was conducted using SPAdes (Holzer and Marz, 2019), while gene annotation was performed with Prokka v1.2.0 (Seemann, 2014). Assembly quality and genome completeness were evaluated using QUAST (Gurevich et al., 2013), and BUSCO (Waterhouse et al., 2018), respectively. Mobile genetic elements, prophage regions, virulence-associated genes, and antimicrobial resistance genes (ARGs) were identified using mobileOG-db v1.1.3 (Brown et al., 2022), PHASTEST v2.0.14 (Wishart et al., 2023), VirulenceFinder v2.0.5 (Malberg Tetzschner et al., 2020), and ResFinder v4.7.2 (Bortolaia et al., 2020), respectively. ARG identification was performed using thresholds of ≥90% sequence identity and ≥60% alignment coverage. CRISPR-Cas systems were analyzed using CRISPRCasFinder (Couvin et al., 2018), whereas bacteriocin-associated genes and other ribosomally synthesized and post-translationally modified peptide clusters were predicted using BAGEL4 (van Heel et al., 2018). Comparative genomic analyses were performed using Proksee (Stothard et al., 2019) and BLASTn (Camacho et al., 2009), and genomic relatedness was further assessed by average nucleotide identity (ANI) analysis using OrthoANI (Lee et al., 2016). Digital DNA–DNA hybridization (dDDH) values were calculated using the Genome-to-Genome Distance Calculator (GGDC 3.0) (Meier-Kolthoff et al., 2022), and whole-genome phylogenetic analysis was performed based on whole-genome sequence comparisons (Olson et al., 2023).

### 2.13 Statistical analysis

All experiments were performed using three independent biological replicates, and each biological replicate was analyzed in triplicate (technical replicates). Data are presented as the mean ± standard deviation (SD). Statistical analyses were performed using one-way analysis of variance (ANOVA) followed by Tukey’s multiple comparison test or Student’s *t*-test, as appropriate. Differences were considered statistically significant at *p* < 0.05.

## 3 Results

### 3.1 Isolation and identification of *L. pentosus* CROT01

Among the LAB isolated from sediment samples collected from the Mekong River, strain CROT01 exhibited the strongest inhibitory activity against *H. pylori* and was therefore selected for further characterization. Phenotypic analysis revealed that the isolate was Gram-positive, rod-shaped, and catalase-negative. MALDI-TOF MS analysis identified the isolate as *Lactiplantibacillus pentosus*, which was subsequently confirmed at the species level through WGS.

### 3.2 Antibacterial activity against *H. pylori*

The antimicrobial activity of *L. pentosus* CROT01 against *H. pylori* reference strains and clinical isolates was evaluated using the agar well diffusion assay. CROT01 exhibited inhibitory activity against all tested *H. pylori* strains, with inhibition zones ranging from 10.67 ± 0.58 to 17.33 ± 1.53 mm (**Table 1**). The strongest antibacterial activity was observed against clinical isolate *H. pylori* 117 (17.33 ± 1.53 mm), followed by *H. pylori* ATCC 700684 (13.33 ± 2.89 mm), *H. pylori* ATCC 43504 (11.67 ± 0.58 mm), and clinical isolate *H. pylori* 004 (10.67 ± 0.58 mm).

**Table 1:** Antibacterial activity of *L. pentosus* CROT01 and its lyophilized cell-free supernatant (LCFS) against *H. pylori* reference strains and clinical isolates determined by agar well diffusion assay, minimum inhibitory concentration (MIC), and minimum bactericidal concentration (MBC).

*H. pylori* strains	Inhibition zone (mm)	MIC (mg/mL)	MBC (mg/mL)
*H. pylori* ATCC 700684	13.33 ± 2.89	50	50
*H. pylori* ATCC 43504	11.67 ± 0.58	12.5	25
*H. pylori* 004	10.67 ± 0.58	50	50
*H. pylori* 117	17.33 ± 1.53	12.5	25

### 3.3 Probiotic characterization *of L. pentosus* CROT01

#### 3.3.1 Gastrointestinal tract tolerance

The GIT tolerance properties of *L. pentosus* CROT01 were evaluated based on its resistance to pepsin, pancreatin, and bile salts. *L. pentosus* CROT01 retained substantial viability following exposure to pepsin at pH 2.0, with a survival rate of 70.14 ± 10.16%, and exhibited high tolerance to pancreatin treatment at pH 8.0, with a survival rate of 99.67 ± 0.58%. However, no viable cells were detected after exposure to 0.3% bile salts (**Table 2**). These findings indicate that *L. pentosus* CROT01 possesses partial tolerance to simulated gastrointestinal conditions.

**Table 2:** Probiotic-related properties of *L. pentosus* CROT01, including gastrointestinal tolerance, cell surface hydrophobicity, and adhesion ability.

Property	Percentage (%)
Pepsin survival rate	85.71 ± 5.11
Pancreatin survival rate	53.33 ± 7.02
0.3% bile salt survival rate	ND
Cell surface hydrophobicity	72.08 ± 2.32
Adhesion to Caco-2 cells	15.66 ± 3.41

ND, not detected.

#### 3.3.2 Cell surface hydrophobicity and adhesion ability

The cell surface hydrophobicity of *L. pentosus* CROT01 toward xylene was high, with a hydrophobicity value of 72.08 ± 2.32%. In addition, the strain exhibited adhesion to Caco-2 cells with an adhesion rate of 15.66 ± 3.41%, indicating favorable adhesion-related properties and the potential to interact with intestinal epithelial surfaces. These characteristics suggest that *L. pentosus* CROT01 may possess the ability to colonize and persist within the gastrointestinal tract, which is considered an important probiotic attribute.

#### 3.3.3 Safety assessment

*L. pentosus* CROT01 exhibited γ-hemolytic activity on blood agar, indicating the absence of hemolytic potential. Antibiotic susceptibility testing demonstrated that the strain was susceptible to erythromycin, tetracycline, ampicillin, and chloramphenicol, while resistance was observed against vancomycin and gentamicin.

### 3.4 MIC and MBC of LCFS against *H. pylori*

LCFS from *L. pentosus* CROT01 exhibited antibacterial activity against all tested *H. pylori* strains (**Table 1**). The MIC values ranged from 12.5 to 50 mg/mL, while the MBC values ranged from 25 to 50 mg/mL. The strongest antibacterial effect was observed against *H. pylori* ATCC 43504 and clinical isolate 117, with MIC and MBC values of 12.5 and 25 mg/mL, respectively. In contrast, *H. pylori* ATCC 700684 and clinical isolate 004 showed lower susceptibility, with MIC and MBC values of 50 mg/mL.

### 3.5 SEM analysis

**Figure 1:**
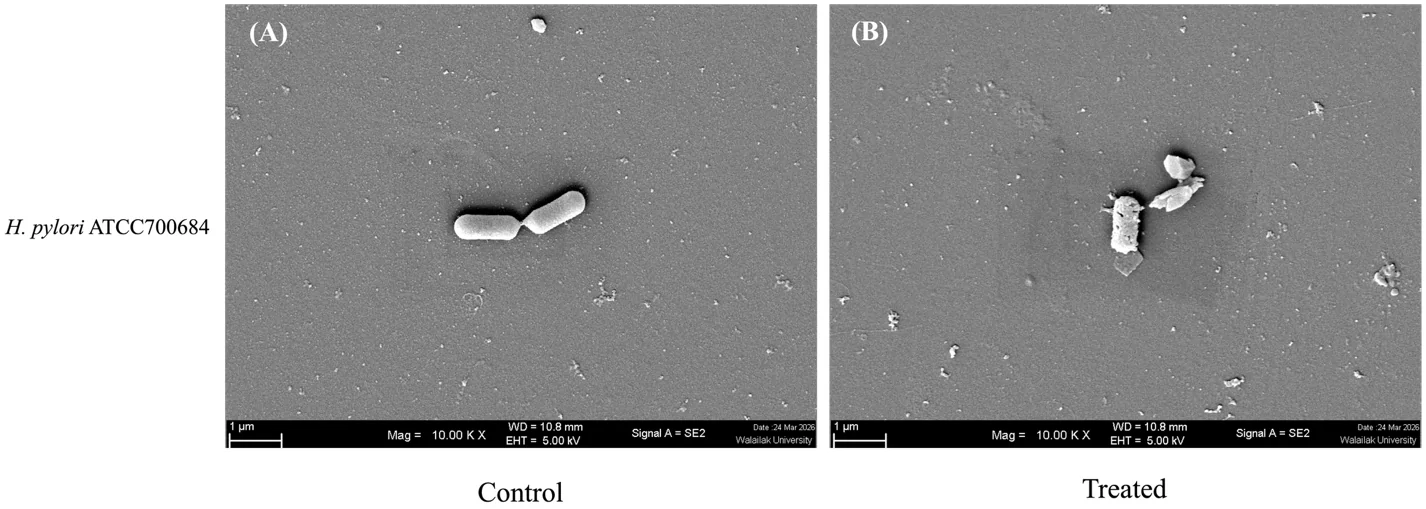
Scanning electron microscopy (SEM) analysis of morphological changes in clarithromycin-resistant *H. pylori* ATCC 700684 following treatment with LCFS from *L. pentosus* CROT01. **(A)** Untreated control cells showing intact helical morphology. **(B)** LCFS-treated cells exhibiting structural damage, including cell shrinkage, membrane disruption, and deformation.

The morphological alterations of clarithromycin-resistant *H. pylori* ATCC 700684 following LCFS treatment were further investigated using SEM. Untreated cells exhibited intact and smooth helical morphology (**Figure 1A**). In contrast, cells treated with LCFS from *L. pentosus* CROT01 displayed pronounced structural damage, including cell shrinkage, surface irregularities, membrane disruption, and deformation (**Figure 1B**). These findings suggest that LCFS induced severe morphological damage to *H. pylori* cells, supporting its antibacterial activity.

### 3.6 Reduction of biofilm formation

**Figure 2:**
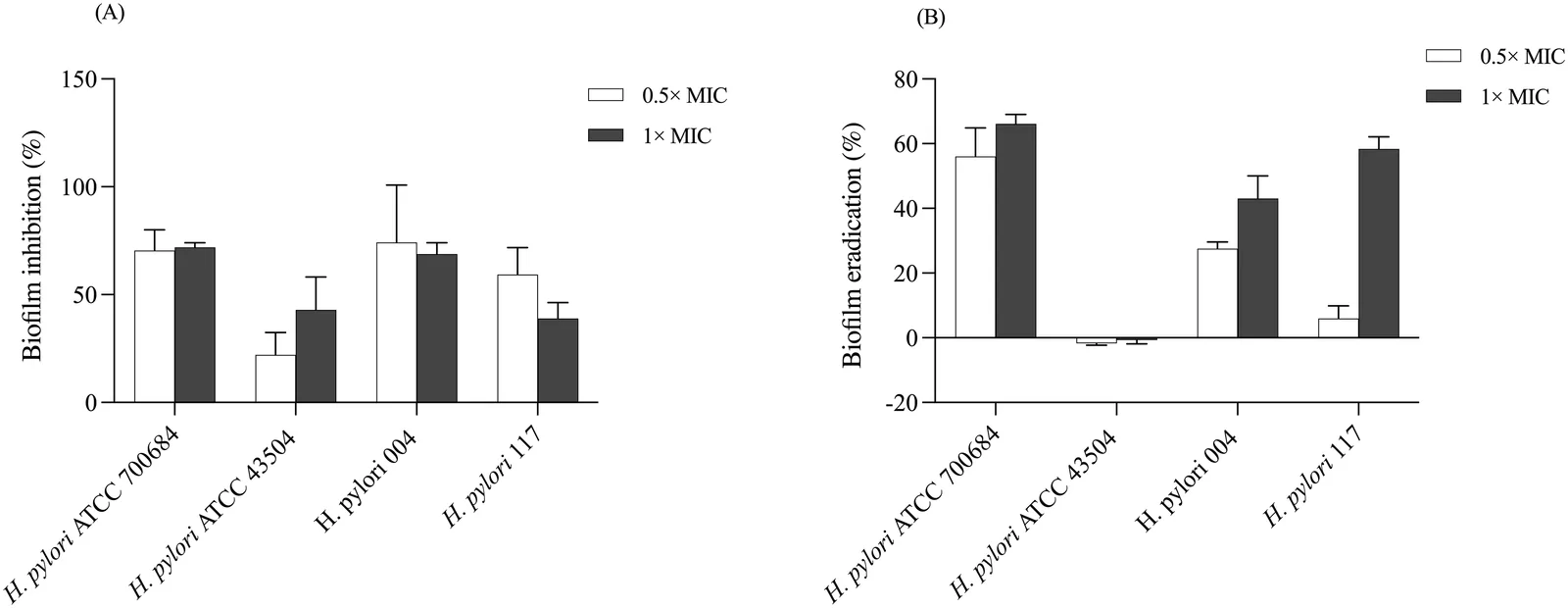
Antibiofilm activity of LCFS from *L. pentosus* CROT01 against *H. pylori* reference strains and clinical isolates. **(A)** Biofilm inhibition activity at 0.5× MIC and 1× MIC concentrations. **(B)** Biofilm eradication activity against preformed biofilms at 0.5× MIC and 1× MIC concentrations. Data are presented as the mean ± standard deviation (SD) of three independent biological experiments, each performed in triplicate. Statistical analysis was performed using one-way ANOVA followed by Tukey’s multiple comparison test.

The antibiofilm activity of LCFS from *L. pentosus* CROT01 against *H. pylori* biofilm formation was determined by the crystal violet assay. As shown in **Figure 2A**, LCFS inhibited biofilm formation in all *H. pylori* strains tested, but the inhibitory effects varied with strain type and concentration. The maximum biofilm inhibition was observed against clinical isolate *H. pylori* 004 (74.13 ± 15.38%) at 0.5× MIC followed by *H. pylori* ATCC 700684 (70.46 ± 6.80%), *H. pylori* 117 (59.28 ± 7.22%) and *H. pylori* ATCC 43504 (22.11 ± 5.99%). At 1× MIC, LCFS showed the highest activity inhibition against *H. pylori* ATCC 700684 with biofilm inhibition rate of 71.98 ± 1.20%, followed by *H. pylori* 004 (68.75 ± 3.78%), *H. pylori* ATCC 43504 (42.97 ± 8.77%) and *H. pylori* 117 (38.95 ± 5.23%). *H. pylori* ATCC 43504 showed enhanced antibiofilm activity at 1× MIC than 0.5× MIC, whereas clinical isolates 004 and 117 showed diminished inhibition. These results show that *L. pentosus* CROT01 LCFS has strain-dependent antibiofilm activity against *H. pylori*.

### 3.7 Activity of LCFS on the eradication of the established biofilms of *H. pylori*

The eradication activity of LCFS from *L. pentosus* CROT01 against preformed *H. pylori* biofilms was evaluated using the crystal violet assay. As shown in **Figure 2B**, LCFS reduced established biofilms in a concentration-dependent manner in most tested strains. At 0.5× MIC, the highest biofilm eradication activity was observed against *H. pylori* ATCC 700684, with an eradication rate of 56.03 ± 5.12%, followed by *H. pylori* 004 (27.51 ± 1.48%) and *H. pylori* 117 (5.94 ± 2.78%). In contrast, negligible eradication activity was observed against *H. pylori* ATCC 43504.

At 1× MIC, LCFS exhibited enhanced eradication activity against most tested strains. The highest eradication effect was observed against *H. pylori* ATCC 700684, with a biofilm eradication rate of 66.13 ± 1.68%, followed by *H. pylori* 117 (58.41 ± 2.12%) and *H. pylori* 004 (43.08 ± 4.03%). No substantial eradication activity was detected against *H. pylori* ATCC 43504. These findings indicate that LCFS from *L. pentosus* CROT01 possesses the ability to disrupt established *H. pylori* biofilms, although the activity was strain dependent.”.

### 3.8 Cell viability by MTT assay

The cytotoxic effects of LCFS from *L. pentosus* CROT01 on AGS and RAW 264.7 cells were evaluated using the MTT assay. LCFS exhibited dose-dependent cytotoxicity toward RAW 264.7 cells, with cell viability decreasing as the concentration increased. Cell viability ranged from 60.01 ± 6.40% at 0.3125 mg/mL to 29.80 ± 1.02% at 20 mg/mL. Moderate cell viability was observed at concentrations between 0.625 and 10 mg/mL, with viability values ranging from 48.41 ± 2.30% to 58.96 ± 4.62%. LC_5_ and LC_10_ values, calculated from dose–response regression analysis, corresponded to 86.93 ± 1.66 µg/mL and 77.70 ± 0.41 µg/mL, respectively. RAW 264.7 cells maintained acceptable viability and were therefore selected for subsequent nitric oxide (NO) inhibition assays. .

Similarly, LCFS treatment also affected AGS cell viability in a concentration-dependent manner. AGS cell viability ranged from 97.21 ± 2.95% at 0.3125 mg/mL to 29.46 ± 2.25% at 20 mg/mL. Higher viability was observed at concentrations between 0.3125 and 5 mg/mL, with viability values exceeding 88%, whereas a marked reduction in viability was observed at concentrations ≥10 mg/mL (**Table 3**).

**Table 3:** Cytotoxic effects of LCFS from *L. pentosus* CROT01 on RAW 264.7 and AGS cells determined by MTT assay.

Conc. (mg/mL)	RAW 264.7 cell viability (%)	AGS cell viability (%)
20	29.80 ± 1.02	29.46 ± 2.25
10	48.41 ± 2.30	69.16 ± 2.62
5	53.41 ± 1.17	88.38 ± 2.94
2.5	50.46 ± 2.61	88.44 ± 1.08
1.25	50.37 ± 3.55	89.65 ± 2.69
0.625	58.96 ± 4.62	92.85 ± 2.22
0.3125	60.01 ± 6.40	97.21 ± 2.95

### 3.9 NO production

**Figure 3:**
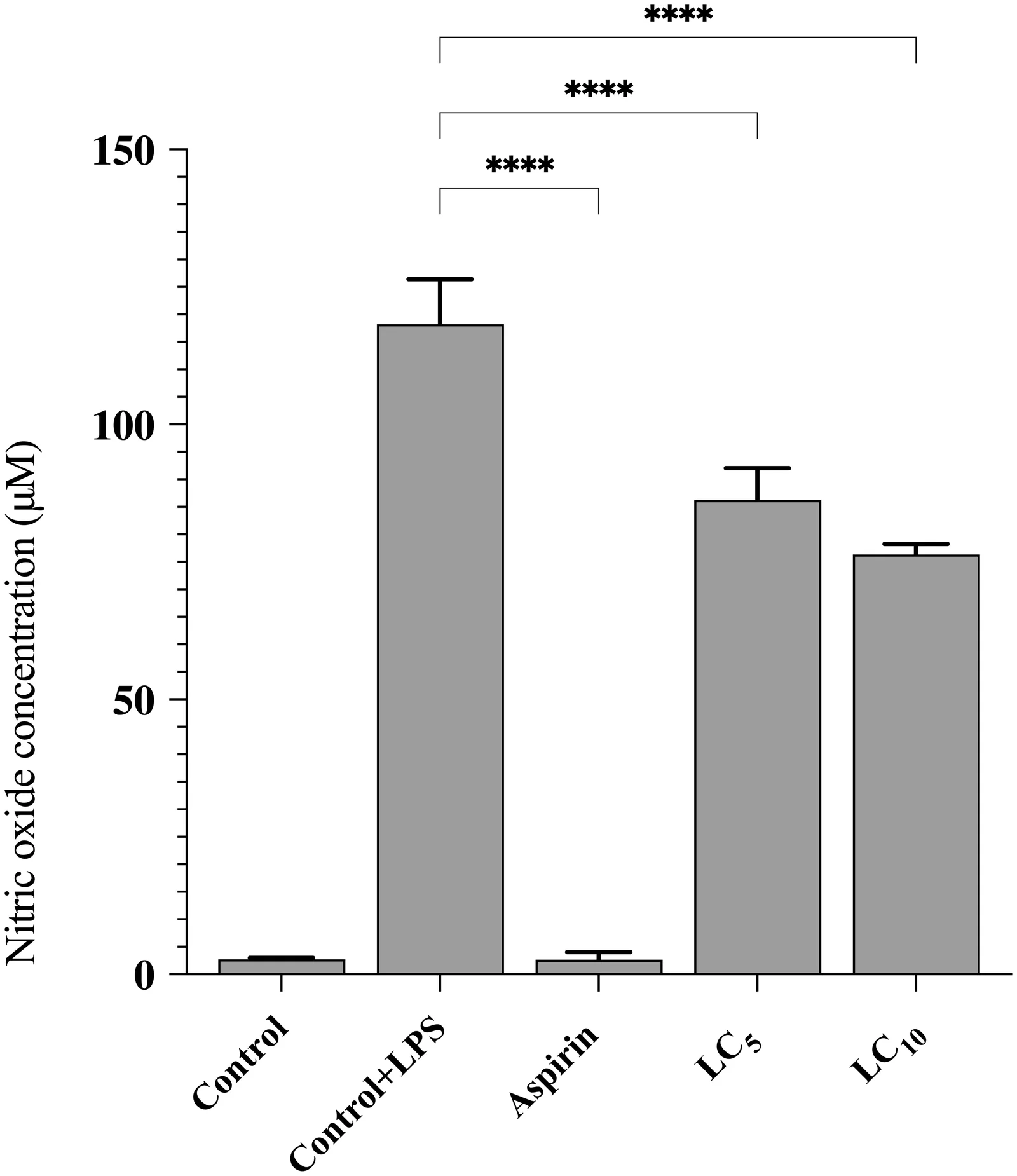
Effects of LCFS from *L. pentosus* CROT01 on nitric oxide production in LPS-stimulated RAW264.7 cells. Data are presented as the mean ± standard deviation (SD) of three independent biological experiments, each performed in triplicate. Statistical analysis was performed using one-way ANOVA followed by Tukey’s multiple comparison test. Differences were considered statistically significant at *p* < 0.05.

NO production in LPS-stimulated RAW 264.7 cells was evaluated to determine the anti-inflammatory activity of LCFS from *L. pentosus* CROT01. As shown in **Figure 3**, stimulation with LPS markedly increased NO production (118.30 ± 4.70 µM) compared with the untreated control group (2.68 ± 0.16 µM). Treatment with aspirin, used as a positive control, significantly reduced NO production to 2.60 ± 0.79 µM. LCFS treatment reduced NO production in LPS-stimulated RAW 264.7 cells. At LC_5_, NO production decreased to 86.22 ± 3.35 µM, whereas treatment at LC_10_ reduced NO levels to 76.36 ± 1.11 µM. A greater reduction in NO production was observed at LC_10_ compared with LC_5_, indicating concentration-dependent anti-inflammatory activity of LCFS from *L. pentosus* CROT01.

### 3.10 Genome features, functional analysis, and antimicrobial substances

**Figure 4:**
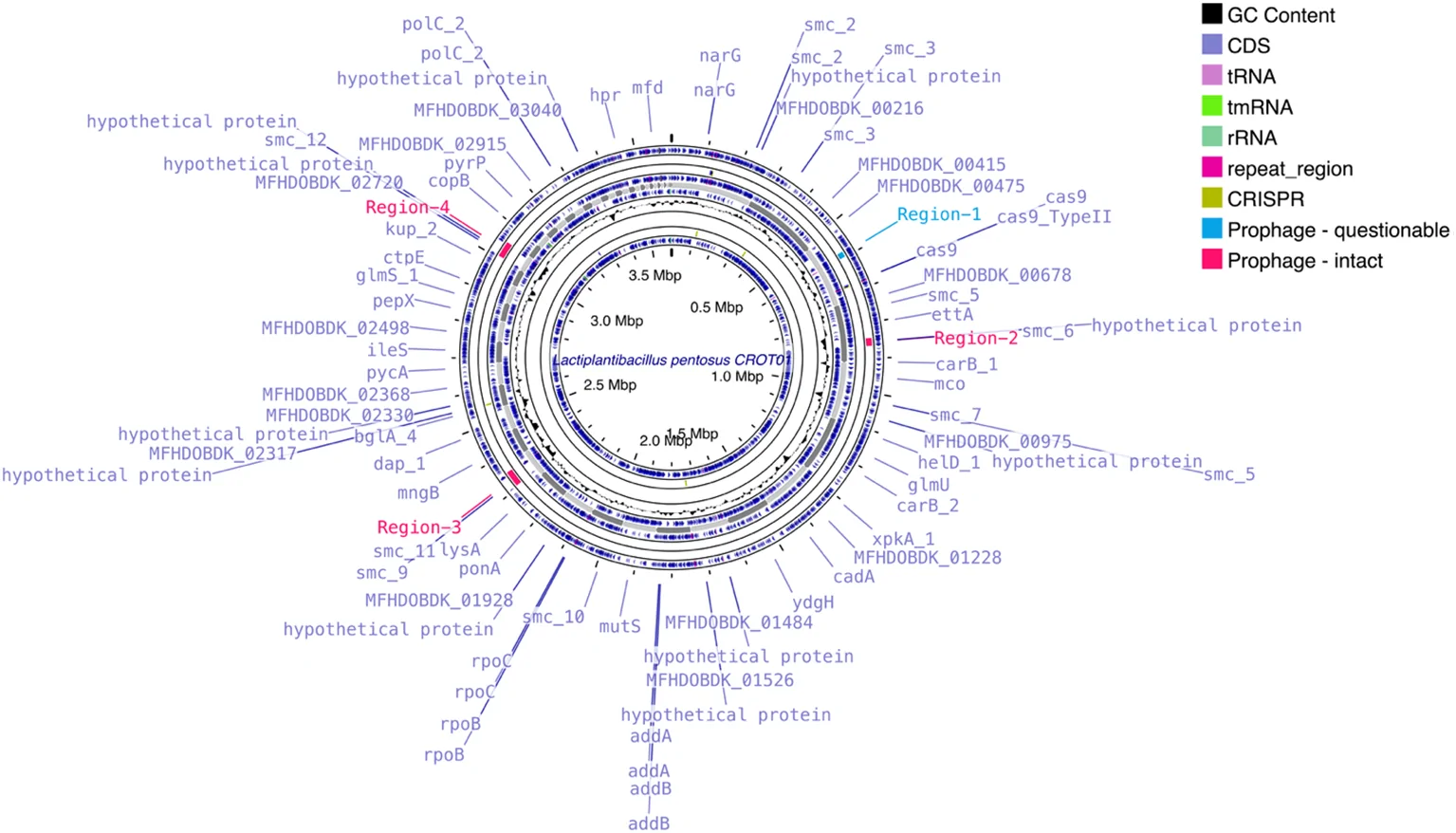
Circular genome map of *L. pentosus* CROT01.

The genomic features of *L. pentosus* CROT01 are summarized in **Table 4**, **Figure 4**. Whole-genome sequencing generated a draft genome of 3,601,066 bp assembled into 52 contigs, with an average GC content of 46.39%. The assembly exhibited an N50 value of 114,495 bp and an L50 value of 10 contigs. Genome annotation predicted 3,239 coding sequences (CDSs), 44 tRNA genes, 3 rRNA genes, and 1 tmRNA gene. In addition, four repeat regions were identified within the genome. No plasmid sequences were detected. The circular genome map (**Figure 4**) illustrates the genomic architecture of *L. pentosus* CROT01, including the distribution of coding sequences, RNA genes, GC content, and GC skew across the genome.

**Table 4:** Genome features of *L. pentosus* CROT01.

Features	*L. pentosus* CROT01
Genome size (bp.)	3,601,066 bp
Contigs	52
GC content (%)	46.39%
N50	114,495
L50	10
Number of CDS	3239
tRNA	44
rRNA	3
tmRNA	1
Repeat Regions	4
Bacteriocin-liked encoding gene	4

Genome quality assessment demonstrated 100% completeness and 0.2% contamination, indicating a high-quality genome assembly. Genome-based taxonomic analysis further confirmed the identity of strain CROT01. Average nucleotide identity (ANI) analysis showed that CROT01 shared 97.82% nucleotide identity with the type strain *Lactiplantibacillus pentosus* DSM 20314, while digital DNA–DNA hybridization (dDDH) analysis yielded a value of 80.50%. Furthermore, whole-genome phylogenetic analysis clustered strain CROT01 within the *L. pentosus* lineage (**Supplementary Figure 1**).

**Figure 5:**
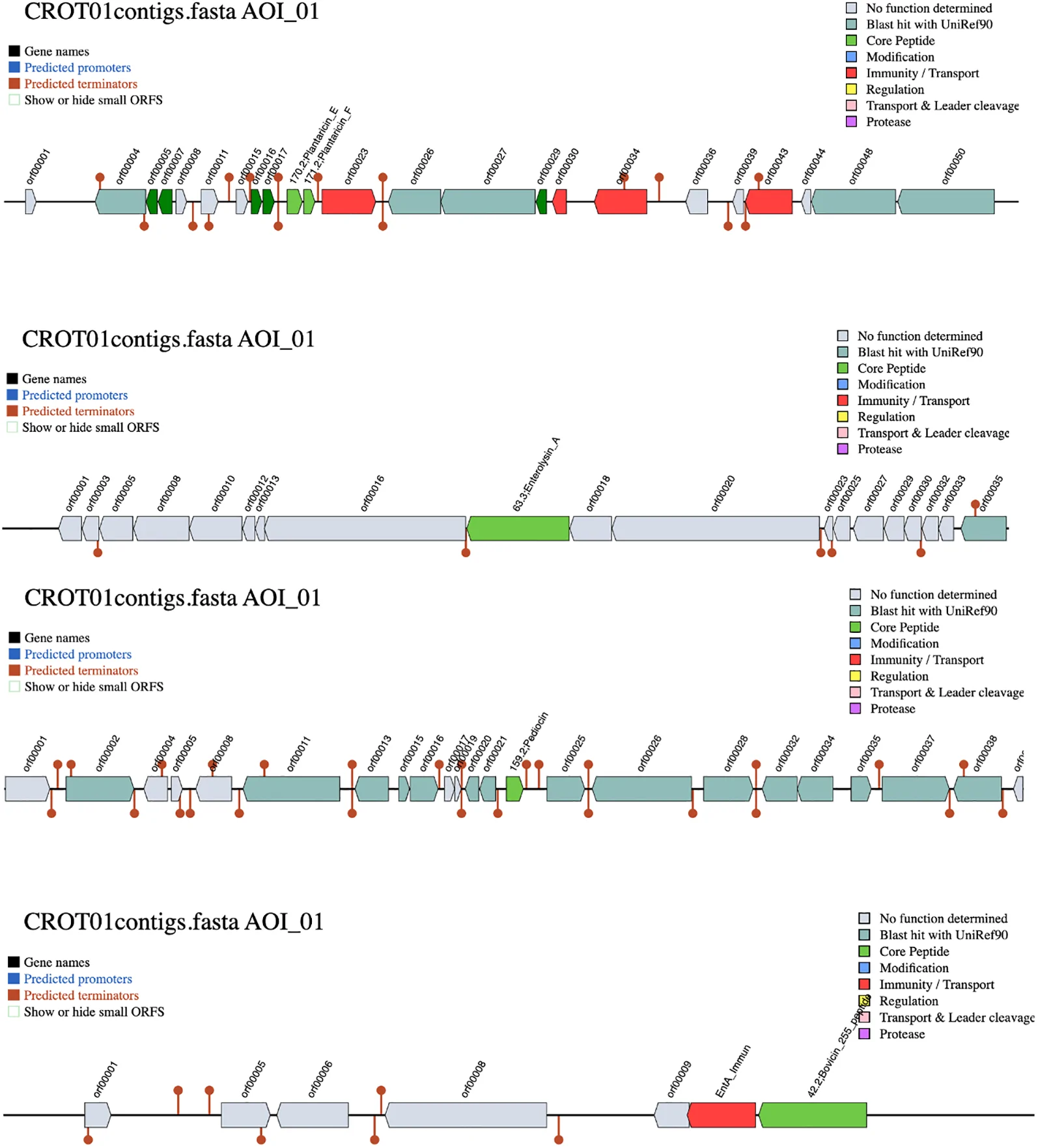
Organization of gene clusters for bacteriocin production in *L. pentosus* CROT01. The cluster comprises genes that encode core peptides, modification enzymes, transport proteins, immune factors, and regulatory proteins, as predicted by BAGEL analysis.

The functional genomic analysis revealed a group of genes associated with probiotic-related traits and environmental stress adaptation (**Supplementary Table 1**). The genome contains genes involved in gastrointestinal survival such as *pbpB, penA, ponA, pbpF* and *pbpX.* The complete F1F0-ATPase operon (*atpA-H*) involved in acid tolerance was identified, bile salt tolerance-related genes (*murE* and *mleS*). We identified several stress-response genes involved in oxidative stress, osmotic adaptation and temperature tolerance, such as *dnaK, dnaJ, clpB, grpE, nox* and *tpx*. Moreover, genes related to biofilm formation and adhesion-related properties were identified, including *luxS, ccpA*, and *dlt* operons. Moreover, bacteriocin-related genes were detected including *plantaricin E*, *plantaricin F, enterolysin A, pediocin*, and *bovicin 255 peptide* (**Figure 5**). The presence of these antimicrobial peptide-encoding genes may contribute to the antibacterial and antibiofilm activities observed against *H. pylori*.

### 3.11 *In silico* safety evaluation

The *in silico* safety assessment of *L. pentosus* CROT01 demonstrated the absence of virulence-associated genes based on analysis using the VFDB. Similarly, no acquired antimicrobial resistance genes were identified, supporting the safety profile of the strain. Genomic analysis further identified prophage-related regions and mobile genetic elements within the genome; however, no transferable resistance determinants or pathogenicity-associated genes were detected. No plasmids were identified in the genome. These findings collectively support the safety and potential applicability of *L. pentosus* CROT01 for probiotic and postbiotic applications.

## 4 Discussion

The increasing incidence of antibiotic-resistant *H. pylori*, especially clarithromycin-resistant strains, has significantly decreased the efficacy of standard eradication regimens, leading to treatment failure and infection recurrence (Elbaiomy et al., 2025). In addition, the ability of *H. pylori* to form biofilms contributes to persistence of the bacteria and antimicrobial tolerance (Graham, 2024). These concerns have increased interest in other therapeutic strategies, such as probiotics, postbiotics, antimicrobial peptides, and bacteriocin-producing bacteria (Yuan et al., 2025). In the present study, LCFS from *L. pentosus* CROT01 isolated from Mekong River sediment displayed antibacterial, antibiofilm, anti-inflammatory, and probiotic-related activities against *H. pylori* confirmed by phenotypic characterization and WGS analysis.

Environmental sediment ecosystems represent dynamic microbial habitats characterized by nutrient fluctuations, oxidative stress, osmotic variation, and intense microbial competition (Liu et al., 2026). Such ecological pressures may favor the evolution of metabolically versatile microorganisms capable of producing diverse extracellular antimicrobial compounds for survival (Liu et al., 2026). In this study, *L. pentosus* CROT01 demonstrated inhibitory activity against both reference strains and clinical isolates of *H. pylori*, including the clarithromycin-resistant strain ATCC 700684. LCFS contains extracellular bioactive metabolites capable of suppressing *H. pylori* viability. Similar inhibitory effects have previously been reported in *Lactobacillus* species, including *L. johnsonii*, *L. gasseri*, *L. reuteri*, and *L. rhamnosus* inhibit *H. pylori.* Probiotic can secrete antibacterial substances such as lactic acid, short-chain fatty acids (SCFAs), hydrogen peroxide, and bacteriocin (Ji and Yang, 2020).

Unlike many previous studies that primarily focused on bacterial growth inhibition, the present study integrated antibacterial, antibiofilm, ultrastructural, anti-inflammatory, and genomic analyses to provide a more comprehensive understanding of the anti-*H. pylori* potential of LCFS from *L. pentosus* CROT01. The structural damage observed in LCFS-treated *H. pylori* cells is consistent with previous reports indicating that metabolites produced by lactic acid bacteria, particularly organic acids and bacteriocin-like peptides, can disrupt membrane integrity and destabilize proton motive force maintenance. Such membrane-targeting mechanisms may be advantageous against antibiotic-resistant bacteria because they differ from the targets of conventional antibiotics and may reduce the likelihood of resistance development (Wan et al., 2023). In addition, bacteriocins such as plantaricins have been reported to interfere with quorum-sensing pathways and biofilm-associated signaling systems, thereby impairing bacterial persistence, colonization, and biofilm maturation within the gastric environment (Wang et al., 2025).

Biofilm formation represents an important virulence mechanism of *H. pylori*, enabling persistent colonization within the gastric mucosa and enhanced tolerance to antibiotics and host immune responses (Ji and Yang, 2020). LCFS from *L. pentosus* CROT01 effectively inhibited biofilm formation and disrupted established biofilms in most tested strains. Strain-dependent differences in susceptibility were observed, with isolate 004 showing the strongest inhibition during biofilm formation, whereas the clarithromycin-resistant strain ATCC 700684 exhibited the greatest susceptibility during biofilm eradication. These differences may reflect variations in extracellular polymeric matrix composition, quorum-sensing regulation, or biofilm maturation among *H. pylori* strains (Hathroubi et al., 2018; Yuan et al., 2025).

Compared with previous studies, LCFS from *L. pentosus* CROT01 exhibited comparable biological activities against *H. pylori*. The antibacterial activity observed in the present study (MIC: 12.5–50 mg/mL; MBC: 25–50 mg/mL) was comparable to or greater than that reported for *Limosilactobacillus fermentum* T0701 (MIC: 25–50 mg/mL; MBC: 50–100 mg/mL) (Sornsenee et al., 2024). In addition, *L. pentosus* SLC13 has been reported to inhibit *H. pylori* growth, urease activity, adhesion, and IL-8 expression in gastric epithelial cells (Thuy et al., 2022), while *L. plantarum* Lp-115 reduced *H. pylori* colonization and gastric inflammation in both AGS cells and a murine model (Shen et al., 2023). Consistent with these findings, LCFS from *L. pentosus* CROT01 eradicated up to 66.13% of preformed biofilms of the clarithromycin-resistant strain ATCC 700684 and reduced nitric oxide production in LPS-stimulated RAW264.7 macrophages. These findings further support the strain-dependent biological activities of probiotic-derived cell-free preparations against *H. pylori*.

WGS identified several bacteriocin-associated genes, including *plantaricin E*, *plantaricin F, pediocin, enterolysin A, and bovicin-related peptides*, which may collectively contribute to the antibacterial and antibiofilm activities observed against *H. pylori*. Plantaricins, particularly class II bacteriocins, are known for their membrane-active properties and have been reported to interfere with membrane integrity and quorum-sensing pathways associated with bacterial persistence and biofilm maturation (Alvarez-Sieiro et al., 2016; Tian et al., 2026). In addition, organic acids produced by LAB may synergistically enhance membrane disruption and promote leakage of intracellular proteins and nucleic acids in *H. pylori* cells (Han et al., 2023). These metabolites may also inhibit urease activity, thereby impairing the ability of *H. pylori* to neutralize acidic environments and reducing its colonization capacity within the stomach (Yuan et al., 2025). Such stress conditions may further induce the transition of *H. pylori* from its characteristic helical morphology into coccoid forms associated with reduced viability and environmental adaptation. Although genome mining identified several bacteriocin-associated genes, including *plantaricin E*, *plantaricin F, enterolysin A, pediocin*, and *bovicin 255 peptide*, the specific bioactive compounds in LCFS were not chemically characterized. Therefore, the proposed mechanisms underlying the antibacterial, antibiofilm, and anti-inflammatory activities remain putative. Future studies involving metabolomic profiling and functional validation are warranted to identify the bioactive compounds responsible for these activities.

The tolerance to simulated gastrointestinal conditions and adhesion-related properties partially supported the probiotic-related characteristics of *L. pentosus* CROT01. The strain demonstrated substantial survival under acidic pH and pancreatin treatment, indicating its ability to tolerate conditions encountered during gastrointestinal transit. Although *L. pentosus* CROT01 exhibited limited tolerance to 0.3% bile salts, the strain showed high cell surface hydrophobicity and moderate adhesion ability toward Caco-2 cells. Cell surface hydrophobicity has frequently been associated with bacterial adhesion and colonization potential, facilitating interactions with epithelial surfaces and competitive exclusion of pathogens. These adhesion-related properties may promote transient interaction with epithelial surfaces and enhance localized delivery of bioactive metabolites within the gastrointestinal environment (Ji and Yang, 2020).

Whole genome sequencing provided mechanistic insight into the experimental phenotypic characteristics observed. Among these, a number of genes related to gastrointestinal tract adaptation and/or tolerance to environmental stress were found, including the entire F1F0-ATPase operon (*atpA-H*) for acid tolerance, along with stress-response genes *dnaK, dnaJ, clpB, grpE, nox* and tpx. These genes are likely to be involved in the resistance of bacteria to acidic and oxidative conditions that are encountered during gastrointestinal transit (Cotter and Hill, 2003; Yang et al., 2026). In addition, genes involved in adhesion and microbial communication such as *luxS, ccpA* and *dlt* operons were identified and may contribute to colonization-related behavior and host interaction (Yang et al., 2021). The genomic and phenotypic findings provide complementary evidence supporting the probiotic- and postbiotic-related characteristics of *L. pentosus* CROT01. However, further transcriptomic and proteomic studies are required to establish direct functional relationships between the identified genes and the observed phenotypes.

Inflammation is a key pathological consequence of *H. pylori* infection and contributes significantly to gastric tissue damage and disease progression (Yuan et al., 2025). Excessive nitric oxide production induced by activated macrophages is closely associated with inflammatory responses and oxidative stress within infected gastric tissues (Ji and Yang, 2020). In the present study, LCFS from *L. pentosus* CROT01 significantly reduced nitric oxide production in LPS-stimulated RAW 264.7 macrophages in a concentration-dependent manner, indicating anti-inflammatory activity. Previous studies have demonstrated that metabolites produced by lactic acid bacteria can suppress inflammatory signaling pathways, including NF-κB and MAPK activation, leading to reduced expression of inducible nitric oxide synthase and decreased production of pro-inflammatory cytokines (Ji and Yang, 2020; Kim and Lee, 2025). Therefore, the reduction in nitric oxide production observed in this study suggests that LCFS from *L. pentosus* CROT01 possesses preliminary immunomodulatory potential, which may contribute to the attenuation of inflammation associated with *H. pylori* infection. Importantly, the NO assay was performed using non-cytotoxic LC_5_ and LC_10_ concentrations that maintained high RAW 264.7 cell viability, suggesting that the observed reduction in nitric oxide production was unlikely to result from the loss of viable macrophages. As the present study was designed as an initial screening to evaluate the anti-inflammatory potential of LCFS, further investigations involving inflammatory cytokines, iNOS expression, and NF-κB signaling are warranted to confirm the underlying immunomodulatory mechanisms.

An important aspect of the present study is the use of LCFS rather than viable probiotic cells. Postbiotic-derived bioactive compounds may provide several translational advantages, including improved formulation stability, easier standardization, reduced risk of horizontal gene transfer, and safer application in immunocompromised individuals (Chen et al., 2025; Pattapulavar et al., 2025). In addition, postbiotic preparations may retain biological activity without requiring bacterial viability, which may improve storage stability and pharmaceutical development (Pattapulavar et al., 2025). These characteristics make postbiotic approaches increasingly attractive as complementary or alternative therapeutic strategies against antibiotic-resistant pathogens.

The safety assessment is an essential step in the development of probiotics and postbiotics (Wala et al., 2023). No virulence-associated or acquired antimicrobial resistance genes were detected, and *L. pentosus* CROT01 exhibited γ-hemolytic activity. Although phenotypic resistance to vancomycin and gentamicin was observed, no acquired antimicrobial resistance genes were identified by ResFinder. This finding is consistent with the intrinsic, non-transferable resistance commonly reported in lactobacilli and is therefore unlikely to pose a risk of horizontal gene transfer (Anisimova et al., 2022). Furthermore, the absence of plasmids and transferable resistance determinants further supports the genomic safety and stability of the strain.

## 5 Conclusion

To the best of our knowledge, this is the first report describing the antibacterial, antibiofilm, anti-inflammatory, and genomic properties of LCFS derived from sediment-associated *L. pentosus* against *H. pylori*. However, the specific metabolites responsible for the observed activities were not chemically characterized, and the precise molecular mechanisms underlying host immunomodulation remain unclear. In addition, the present study was conducted primarily under *in vitro* conditions. Further investigations involving metabolomic profiling, purification of active antimicrobial compounds, transcriptomic analysis, and *in vivo* validation are warranted to better elucidate the therapeutic potential of LCFS from *L. pentosus* CROT01. Despite these limitations, the findings support the potential application of LCFS from *L. pentosus* CROT01 as a promising non-antibiotic strategy against antibiotic-resistant *H. pylori* infection.

## Data Availability

The data presented in the study are deposited in the NCBI GenBank repository, accession number JCBOZV000000000. BioProject: PRJNA1502567. BioSample: SAMN60504956. Organism: *Lactiplantibacillus pentosus* CROT01.
