# Supplementary material for: Genomic characterization and biological activities of cell-free supernatant from Lactiplantibacillus pentosus CROT01 against clarithromycin-resistant Helicobacter pylori
Source: Front Cell Infect Microbiol. 2026 Aug 12;16:1882551. doi: 10.3389/fcimb.2026.1882551 (PMC13506446; doi:10.3389/fcimb.2026.1882551)
Supplement: Supplementary file 1 [file Table1.docx]

**Supplementary table 1.** Genes associated with probiotic functions of *L. pentosus* CROT01.

| **Function** | **Gene** | **CROT01** |
| --- | --- | --- |
| Gastrointestinal tract survival | *pbpB* | + |
|  | *penA* | + |
|  | *pbpE* | - |
|  | *ponA* | + |
|  | *pbpF_1* | + |
|  | *pbpF_2* | + |
|  | *pbpX* | + |
|  | *pbp* | - |
| Acid tolerance | *nhaK_2* | + |
|  | *atpA* | + |
|  | *atpF* | + |
|  | *atpG* | + |
|  | *atpB* | + |
|  | *atpD* | + |
|  | *atpH* | + |
|  | *atpE* | + |
| Bile salt tolerance | *murE* | + |
|  | *mleS* | + |
| Temperature tolerance | *cspB* | - |
|  | *cspLA* | + |
|  | *csp* | + |
|  | *hrcA* | + |
|  | *dnaJ* | + |
|  | *dnaK* | + |
|  | *clpC_1* | + |
|  | *clpB* | + |
| Osmotic shock tolerance | *grpE* | + |
|  | *gbuA* | - |
|  | *gbuC* | - |
|  | *gbuB* | - |
|  | *opuCD* | + |
|  | *opuCC* | + |
| Oxidative stress survival | *hslO* | + |
|  | *nox_2* | + |
|  | *nox_1* | + |
|  | *tpx* | + |
|  | *npr* | + |
| Cell wall formation | *murA1* | + |
|  | *epsH_2* | + |
|  | *ykoT_1* | + |
|  | *tagE* | + |
|  | *dltC* | + |
|  | *dltA* | + |
|  | *dltD* | + |
|  | *dltC* | + |
| Biofilm formation | *ywqC* | - |
|  | *luxS* | + |
|  | *desR* | + |
|  | *ccpA_2* | + |
|  | *brpA_2* | - |
|  | *brpA_4* | - |
|  | *brpA_3* | - |
| Vitamin synthesis | *btuD_14* | - |
|  | *btuD_14* | - |
|  | *btuD_2* | + |
|  | *btuD_8* | + |
|  | *btuD_13* | - |
|  | *btuD_4* | + |
|  | *btuD_15* | - |
|  | *btuD_5* | + |
|  | *btuD_9* | + |
|  | *btuD_12* | - |
|  | *btuD_11* | - |
|  | *btuD_7* | + |
|  | *btuD_6* | + |
|  | *btuD_1* | + |
|  | *btuD_3* | + |
| Bacteriocin | *Plantaricin_E* | + |
|  | *Plantaricin_F* | + |
|  | *Enterolysin_A* | + |
|  | *Pediocin* | + |
|  | *Bovicin_255_peptide* | + |

**Supplementary figure 1.** Whole-genome phylogenetic tree showing the taxonomic position of *Lactiplantibacillus pentosus* CROT01 among closely related *Lactiplantibacillus* species.
